# A Pipeline Defect Instance Segmentation System Based on SparseInst

**DOI:** 10.3390/s23229019

**Published:** 2023-11-07

**Authors:** Niannian Wang, Jingzheng Zhang, Xiaotian Song

**Affiliations:** 1 School of Water Conservancy and Transportation, Zhengzhou University, Zhengzhou 450001, China; wnnian@zzu.edu.cn (N.W.); zjz981026@163.com (J.Z.); 2 School of Engineering and Technology, China University of Geosciences (Beijing), Beijing 100083, China

**Keywords:** deep learning, image segmentation, pipeline defects, data augmentation

## Abstract

Deep learning algorithms have achieved encouraging results for pipeline defect segmentation. However, existing defect segmentation methods may encounter challenges in accurately segmenting the complex features of pipeline defects and suffer from low processing speeds. Therefore, in this study, we propose Pipe-Sparse-Net, a pipeline defect segmentation system that combines StyleGAN3 to segment the complex forms of underground drainage pipe defects. First, we introduce a data augmentation algorithm based on StyleGAN3 to enlarge the dataset. Next, we propose Pipe-Sparse-Net, a pipeline segmentation model based on SparseInst, to accurately predict the defect regions in drainage pipes. Experimental results demonstrate that the segmentation accuracy of this model can reach 91.4% with a processing speed of 56.7 frames per second (FPS). To validate the superiority of this method, comparative experiments were conducted against Yolact, Condinst, and Mask R-CNN, and the model achieved a speed improvement of 45% while increasing the accuracy by more than 4%.

## 1. Introduction

Urban drainage pipelines are essential components of urban infrastructure and resemble the vascular system of a city. With the accelerated urbanization process in China, the total length of urban drainage pipelines is increasing at a rate of 5.5–10% per year, reaching 802,000 km [1]. These pipelines provide great convenience to human communities but require regular inspection, cleaning, and maintenance. Various functional defects can occur in drainage pipelines, such as sludge deposition, pipe-wall scaling, and interface leakage. These issues can result in poor drainage, reduced pipeline functionality, and localized flooding or environmental pollution. To ensure the smooth operation and safety of drainage pipeline facilities, detection techniques are required to understand the functional and structural conditions inside pipes [2], guiding maintenance and management efforts.

Currently, visual inspection techniques such as Closed-Circuit Television (CCTV) [3] and QuickView (QV) [4] are primarily used for underground drainage pipeline inspection and collecting image or video data. With the widespread development and application of visual inspection technologies used in infrastructure such as CCTV robots and QV, large volumes of inspection images and videos are generated. Manual classification and interpretation of these visual data are inefficient and inaccurate [5]. Many researchers have proposed automated defect recognition techniques based on computer vision and image processing technologies, which show promise in pipeline defect identification and analysis.

### 1.1. Related Work

#### 1.1.1. Traditional Computer Vision and Image Processing Techniques

Traditional computer vision techniques have been employed for the automated interpretation of CCTV images, requiring extensive image preprocessing and the design of complex feature extractors for tasks such as feature extraction and classification of sewage pipeline images [6,7,8]. Li Hongyi [9] proposed a three-dimensional pipeline reconstruction method based on DWGDirect technology designed to extract entity information from DWG format files. Huynh et al. [10] proposed a sewage pipeline detection system based on 3D stereovision. Their method achieved stereovision through calibration, rectification, and correspondence and used a bilateral edge evaluation algorithm to detect small cracks in pipelines. They also employed a constrained sliding window algorithm to improve search speed. However, traditional computer vision techniques face several challenges, including handling low-resolution and noisy videos, dealing with image distortion and structural motion [11], and being influenced by lighting and shooting distances [12]. In the field of pipeline defect recognition, traditional computer vision techniques encounter two main issues. First, they require the design of complex feature extractors that are specific to the task at hand. Second, the preparation of training datasets involves extensive image preprocessing, which makes the training process cumbersome. Furthermore, current research primarily focuses on identifying and detecting individual defects, such as cracks, while the automatic recognition and localization of other common defects, such as misalignments and obstructions, remain limited.

#### 1.1.2. Deep Learning Techniques

In recent years, deep learning algorithms [13,14,15] have emerged for use in image classification. Deep learning mimics the neural network mechanisms of the human brain to recognize and analyze data such as digital images, text, and speech. This involves combining low-level features of data to form high-level image attribute features that may not be easily discernible. In contrast to traditional image recognition methods that require manual feature design and extraction from raw data, deep learning enables the representation of a large amount of raw data with accurate latent features. This representation requires specialized knowledge and extensive work experience using traditional methods. Traditional image recognition methods are complex and inefficient, whereas deep learning technology provides an effective solution to these challenges.

In 2020, Wang and Cheng proposed an improved neural network called DilaSeg-CRF [16], which utilizes dilated convolutions and multiscale techniques to generate feature maps with higher resolutions, thereby enhancing the accuracy of pipeline defect segmentation. In the same year, Pan et al. [17] introduced a novel semantic segmentation network called PipeUNet for drainage pipeline defect segmentation, using U-Net as the backbone network. It achieved high-speed processing of CCTV images at a rate of 32 fps. Qianqian et al. [18], in 2021, proposed a method based on DeepLabv3+ for automatic pixel-level segmentation and severity quantification of sewer defects. Ma et al. [19] proposed a automatic intelligent detection and tracking system for road cracks composed of a Generative Adversarial Network (PCGAN) and a crack detection and tracking network called YOLO-MF in 2022. The improved algorithm achieved the highest accuracy of 98.47%. In the same year, He et al. [20] proposed an image segmentation method based on deep convolutional neural networks, which achieved pixel-level segmentation of defect regions while classifying pipeline defects. Li et al. [21], also in 2022, introduced a new instance segmentation model called Pipe-SOLO, which features an efficient backbone structure (Res2Net-Mish-BN-101) and an enhanced BiFPN network for underground pipeline defect segmentation.

Deep learning technology provides an important solution by eliminating the need for manual feature engineering. Instead, a large amount of raw data are directly input into deep neural networks and then undergo extensive nonlinear transformations based on specific algorithms to extract high-level abstract features. Deep neural networks achieve learning of highly complex functions by thoroughly transforming and combining raw data. Therefore, the prerequisite for applying deep learning methods is to train the neural network using a substantial amount of data, enabling the network to learn generalizable features. Various types of defects in drainage pipelines exhibit distinct visual characteristics, which is advantageous for the automatic recognition and extraction of features by deep learning networks. Additionally, the widespread use of CCTV photography techniques for drainage pipelines allows the acquisition of a large dataset of defect images within a short period. Deep learning technology can be successfully applied to defect recognition in drainage pipelines. The significant advantages of applying deep learning to intelligent recognition of underground pipeline defects include automatic feature learning, replacement of manual feature engineering, high efficiency and stable performance, and the ability to leverage parallel computing using graphics processing units (GPUs) for accelerated processing, resulting in significantly improved recognition speed compared to typical image processing techniques. Furthermore, deep learning can optimize feature representation by leveraging joint automatic classifiers, greatly enhancing performance. In 2018, Myrans [22] proposed a cascaded model based on a SVM radio-frequency classifier for automatic pipeline fault detection, which significantly reduced false alarm rates. Finally, deep learning can benefit from the enormous advantages offered by big data, thereby improving its scalability.

### 1.2. Contributions

**Figure 1:**
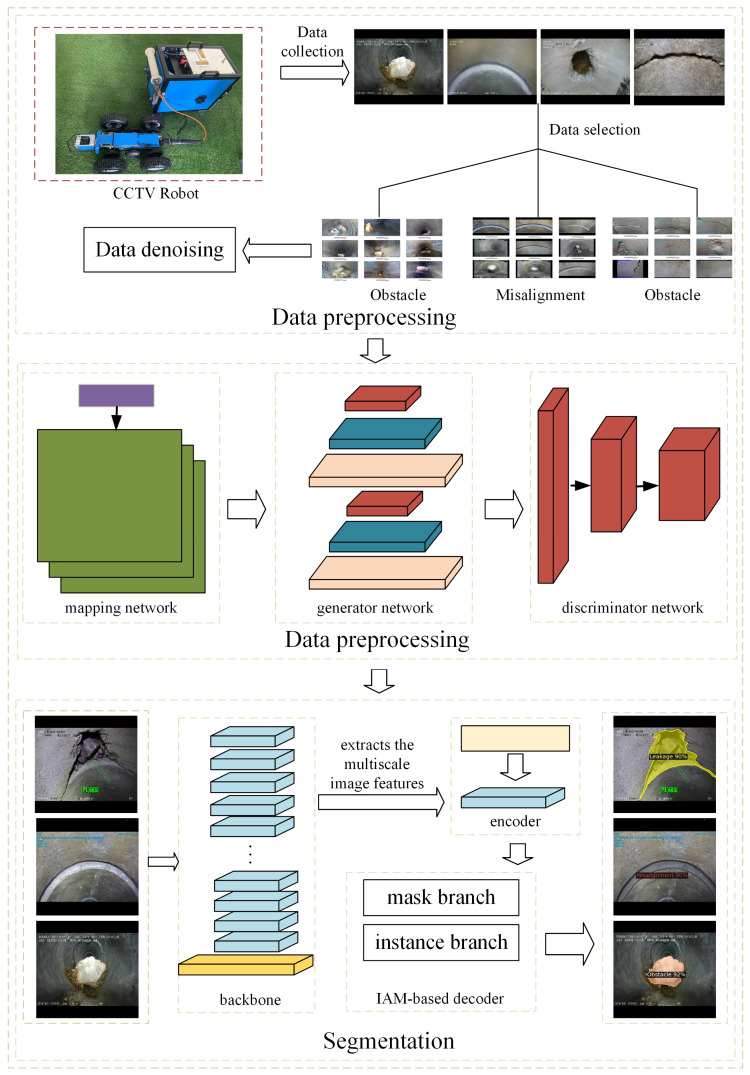
The framework of intelligent pipeline defect segmentation system.

As shown in Figure 1, a new intelligent pipeline defect segmentation system was proposed to further improve the accuracy and speed of underground drainage pipeline defect segmentation. The main innovations of this study are summarized as follows.

1. To address the issue of insufficient pipeline defect data, a clear image generation network for drainage pipeline defects, called Pipe-Gan-Net, is established based on StyleGAN3. This network was used to increase the number of new defect images;
2. To improve the accuracy and speed of pipeline defect segmentation, a pipeline segmentation model called Pipe-Sparse-Net is proposed based on SparseInst. This model accurately predicts the regions of drainage pipeline defects;
3. To further enhance the detection speed, an acceleration module called TensorRT is applied to the segmentation model.

The remainder of this paper is organized as follows: Section 2 introduces the creation of the dataset for pipeline defect segmentation, including data preprocessing, data augmentation, and the instance segmentation model. Section 3 presents the experimental research on the training and evaluation of image preprocessing and segmentation networks. It also discusses the impact of data augmentation and TensorRT on segmentation. Section 4 summarizes the findings of the study and discusses future work.

## 2. Methodology

In this study, we propose Pipe-Sparse-Net, a pipeline defect segmentation model combined with StyleGAN3, to segment complex forms of defects in underground drainage pipelines. First, images collected from the CCTV pipeline robots were selected and resized to 512 × 512 pixels. StyleGAN3 was used to pre-process the original images and generate a large number of defect images. Both the original and generated images were input into the underground drainage pipeline defect segmentation network based on SparseInst. When defects were detected, the network generated defect prediction masks based on SparseInst to indicate the actual defects present in the images. Subsequently, the topological features of the defect regions were extracted based on the accurately predicted defect masks.

### 2.1. Drainage Pipeline Defect Image Generation Network Pipe-Gan-Net

This study proposes a data augmentation method based on StyleGAN3 [23] that enhances various pipeline defect images without replacing the original dataset, thereby improving the performance of the segmentation model. StyleGAN3 addresses the issue of image coordinates and feature entanglement in StyleGAN2, achieving true invariance in image translation, rotation, and other transformations and significantly improving the image synthesis quality. The model consists of a generator network and a discriminator network, both of which are deep neural networks utilizing Leaky ReLU as the activation function. The generator network employs a CNN and comprises a mapping network and a synthesis network consisting of eight fully connected layers. The objective of this network is to encode the input random noise vector Z into an intermediate vector W, where the different elements of the intermediate vector control different visual features. These visual features are used to determine the style of the generated pipeline defect images. In the mapping network, Fourier features are utilized instead of constant inputs, noise inputs are removed (the position information of features is solely derived from the coarse features of the previous layers), the network depth is reduced to 14 layers (previously 18 layers), mixing regularization and path length regularization are disabled, and simple normalization is applied before each convolution.

The synthesis network consists of 16 convolutional layers, 14 upsampling layers, and 14 downsampling layers, which are responsible for generating pipeline defect images. In the synthesis network, the network depth is reduced, mixing regularization and path length regularization are disabled, and simple normalization is applied before each convolution. Non-linear filtering is improved, and the entire upsample–LReLU–downsample process is implemented in a custom CUDA kernel. To achieve rotational invariance in the network, the kernel size of all layers was varied from 3 × 3 to 1 × 1. The quantity of the feature map was doubled to compensate for the reduced feature capacity.

**Figure 2:**
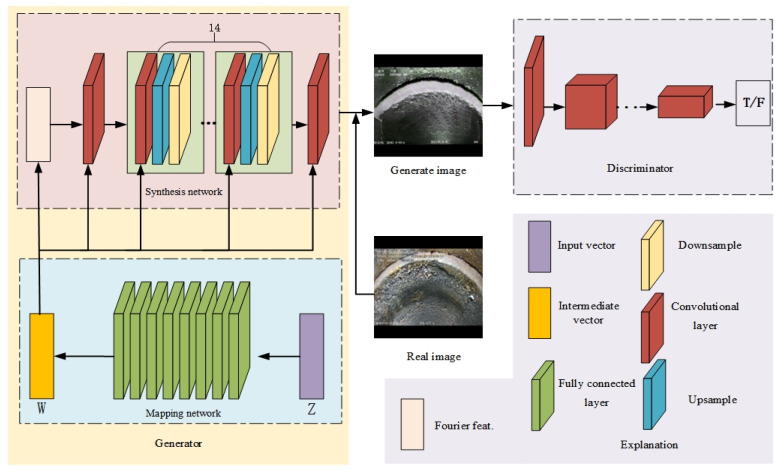
Pipeline defect data augmentation model based on StyleGAN3.

The generated data from the generator network aim to be as close as possible to real data, allowing us to effectively augment the research data. The structure of our model is illustrated in Figure 2.

In this study, after preprocessing, the numbers of images in the dataset for cracks, obstacles, and holes were 499, 315, and 472, respectively. After applying this data augmentation method, the number of images for the three types of damage mentioned above was 1497, 945, and 1416.

### 2.2. Drainage Pipeline Defect Image Segmentation Network Pipe-Sparse-Net

Recently, deep learning-based segmentation algorithms have been divided into two categories. The first category comprises region-based methods, such as Faster R-CNN [24], which detects objects and obtains bounding boxes. RoIPooling [24] or RoI Align [25] is then applied to extract region features for pixel-wise segmentation. Mask R-CNN [25], as a representative method, extends Faster R-CNN by adding a mask branch to predict object masks. The second category includes center-based methods. YOLACT [26] generates instance masks by combining mask coefficients and prototype masks. MEInst [27] and CondInst [28] expand upon FCOS [29] by predicting encoding mask vectors or mask kernels of dynamic convolutions [8]. SOLO [30,31], as a detector-free method, still performs object localization and recognition based on the centers and generates mask kernels. SparseInst [32] uses sparse instance activation maps to represent objects in a simple and efficient manner.

SparseInst outputs a fixed-size set of prediction results. To address the end-to-end training problem, we represent label assignment as bipartite matching [33] in Equation (1). We propose a pairwise matching score C(i,k) based on the Dice coefficient, which is determined by the classification score and Dice coefficient of the segmentation mask, to match *i* predictions with k ground-truth instances.

(1) $$C(i,k)=p_{i,c_{k}}^{1-\alpha }\cdot \mathrm{DICE}(m_{i},t_{k}{)}^{\alpha }$$

where *α* is a hyperparameter used to balance the influence of classification and segmentation and is empirically set to 0.8. ck represents the class label of the kth ground truth instance, and $p_{i}$, ck denotes the probability of the ith prediction belonging to class $c_{k}$. mi and $t_{k}$ are the masks of the ith prediction and kth ground-truth instance, respectively. The Dice coefficient is defined by Equation (2).

(2) $$\mathrm{DICE}(m,t)=\frac{2\sum_{x,y}m_{xy}\cdot t_{xy}}{\sum_{x,y}m_{xy}^{2}+\sum_{x,y}t_{xy}^{2}}$$

where $m_{xy}$ and $t_{xy}$ represent the pixels at coordinates (*x, y*) in the predicted mask m and the ground-truth instance mask t, respectively. Then, we utilized the Hungarian algorithm [34] to find the optimal matching between K ground-truth instance objects and N predicted objects. The training loss is defined in Equation (3) and includes the classification loss, object prediction loss, and segmentation loss.

(3) $$L=\lambda_{c}\cdot L_{cls}+L_{mask}+\lambda_{s}\cdot L_{s}$$

where is the focal loss [35] for object classification, is the mask loss, and is the binary cross-entropy loss for IoU-aware objectness. Considering the severe imbalance between the background and foreground in full-resolution instance segmentation, we employed a hybrid mask loss that combined the Dice loss [36] and pixel-wise binary cross-entropy loss for segmentation masks, as shown in Equation (4).

(4) $$L_{mask}=\lambda_{dice}\cdot L_{dice}+\lambda_{pix}\cdot L_{pix}$$

where and are the Dice and binary cross-entropy losses, respectively, and and are the corresponding coefficients.

**Figure 3:**
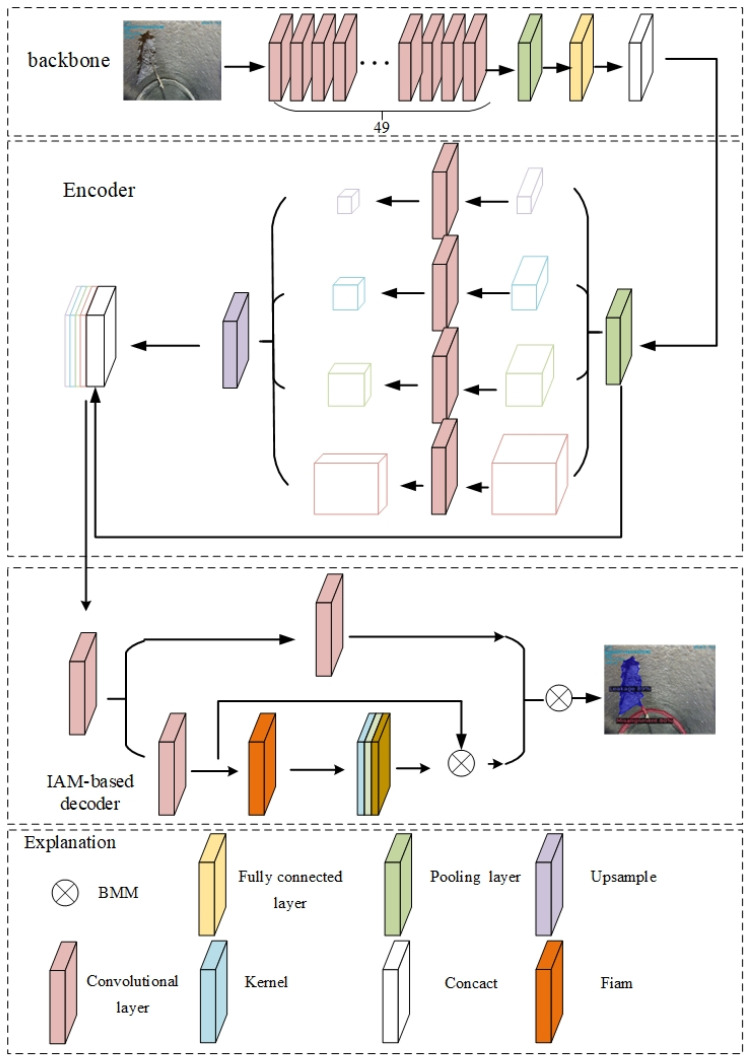
Architecture of Pipe-Sparse-Net model.

We propose a deep learning model called Pipe-Sparse-Net for the segmentation of defects in underground drainage pipes. It consists of a backbone network, instance context encoder, and decoder based on the Iterative Anchor Matching (IAM) algorithm. The backbone network utilizes ResNet50 [37] to extract multiscale features from the input image. The instance context encoder is connected to the backbone network to enhance contextual information and fuse multiscale features. These features are then fed into the subsequent decoder based on IAM to generate instance activation maps, highlighting foreground objects for classification and segmentation. Detailed network parameters are shown in Figure 3.

Pipe-Sparse-Net utilizes a method that can output sparse instance activation maps to represent each target object, highlighting the informative regions of the foreground objects. The highlighted regions are then aggregated to obtain instance-level features for subsequent segmentation. Based on bipartite matching, the model predicts objects in a one-to-one manner, avoiding the need for subsequent non-maximum suppression (NMS), thus improving both the segmentation accuracy and speed of the model.

## 3. Experiments and Analysis

### 3.1. Experimental Settings

This study utilized the Ubuntu 18.04 system as a platform for training the deep learning model. Specifically, the system was equipped with a 12 GB GeForce RTX3080Ti GPU and 64 GB RAM I9 9900k, providing high-performance computing support. Python 3.8 was used as the primary programming language, and the detectron2-v0.6 framework was employed for training, validation, and testing of the deep learning model.

### 3.2. Pipeline Dataset

**Figure 4:**
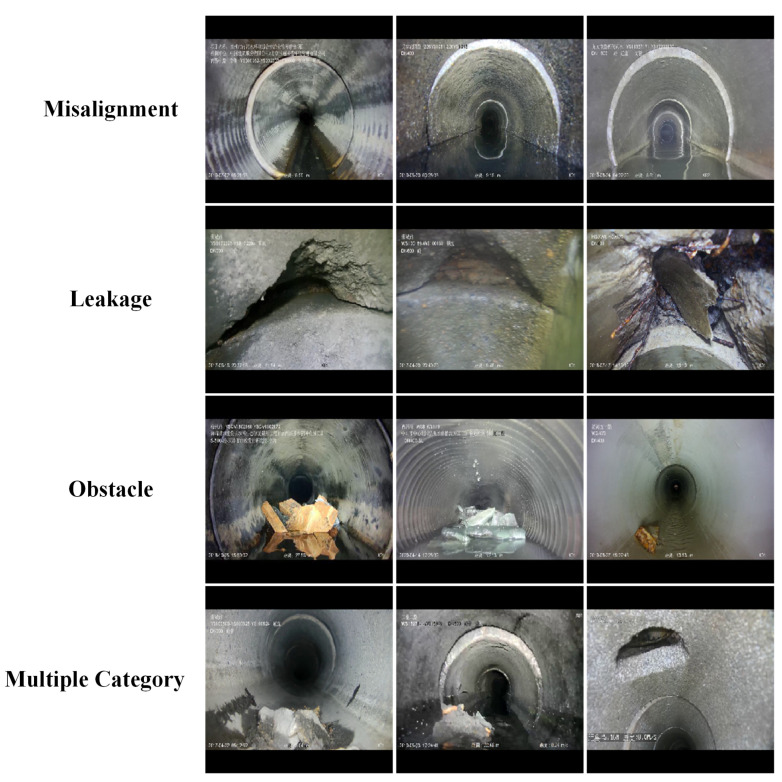
Example of pipeline defects.

In this study, CCTV robots collected images of sewer pipe defects in Zhengzhou and Tianjin. The original images were strictly selected by professional inspectors to enhance the image clarity and ensure a balanced number of defects in each category. In addition, the perspectives varied, including front and side views. A total of 1585 original images containing three types of flaws (misalignments, obstructions, and cracks) were used for real-time segmentation. Misalignments were considered lateral deviations of circular pipe joints, whereas cracks were treated as fractures caused by external pressure on the pipes, including fissures and pits. In some images, there were multiple flaws in different categories, making segmentation challenging. Examples of pipe defect images are shown in Figure 4. The LabelMe software was used to label the data using mask annotations and the LableMe version is 4.5.13. LabelMe is an open-source semi-automatic image annotation tool that supports the creation of object segmentation datasets by importing annotated images and manually tracing the objects using closed curves. Images were randomly divided into training, validation, and test sets in a 6:2:2 ratio, which is a common segmentation ratio, especially when the dataset is small. These three datasets do not contain the same images, ensuring that the trained model can be generalized to new images. Finally, the information was converted to the standard COCO2017 format [38]. There were 1585 defects in the original images. Misalignments were the most numerous and cracks were the rarest. The distribution ratios of the three categories in the training, validation, and test sets were similar to the 6:2:2 image split ratios shown in Table 1.

### 3.3. Model Training and Validation

#### 3.3.1. Parameter Settings

The goal of training deep learning models is to continuously adjust and optimize the model parameters through repeated training on massive datasets to minimize the overall loss. Optimization is performed using stochastic gradient descent, which is one of the most commonly used optimization methods in deep learning models and outperforms traditional optimizers. The learning rate controls how quickly the gradient descent moves, thereby affecting the speed of model training and learning. If the learning rate is too high, the loss value increases and the model does not converge. If it is too small, the loss value barely changes and the model learns very slowly. The validation results of the models with different hyperparameter combinations are presented in Table 2. Ultimately, the learning rate, momentum, and weight decay were set to 5 × 10^−4^, 0.9, and 5 × 10^−4^.

During each training iteration, the images were fed into the network in batches for processing, with each batch containing four images. This reduced GPU memory requirements and improved training efficiency. The images were randomly shuffled before each epoch to prevent overfitting.

**Figure 5:**
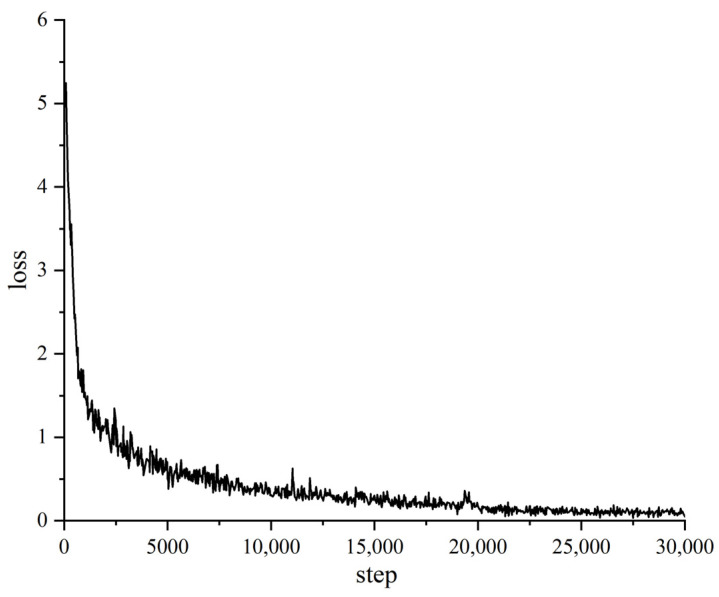
Loss curve of model training.

**Figure 6:**
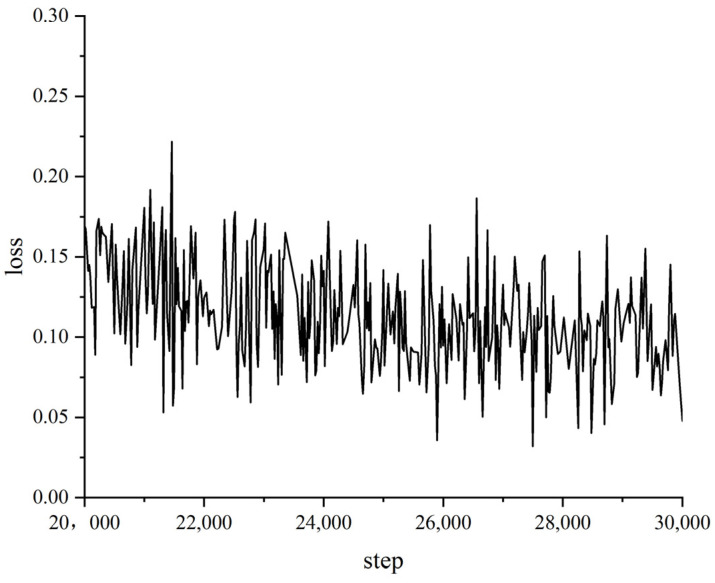
Local loss curve when converging.

After completing the experimental environment setup and parameter initialization, we started training the Pipe-Sparse-Net model. The number of training iterations has a big impact on model accuracy and learning time. Fewer iterations lead to shorter training times but may be unable to minimize the training loss, resulting in lower accuracy. More iterations mean the training loss can be minimized but lead to excessively long training times, wasting resources. The loss is a metric that measures the difference between the predicted outputs of a model and the actual labels. The loss value is typically a scalar that indicates the performance of the model. Since the loss value is a numerical quantity, it does not have a specific unit. The total number of iterations was set to 30,000, with the loss value on the training set saved every 20 iterations. The loss function curve is shown in Figure 5. After 22,000 iterations, the proposed model achieved the highest accuracy of 91.4%. The model has strong generalization capability. When the number of iterations is small, the model has not fully learned the features of the defects and is in the underfitting stage. When the number of iterations is too large, the model learns meaningless noise, decreasing accuracy, which is the overfitting stage. From the loss function curve, it can be seen that after 22,000 iterations, the loss value tends to converge and reaches a local optimal solution. The local loss function curve is shown in Figure 6.

**Figure 7:**
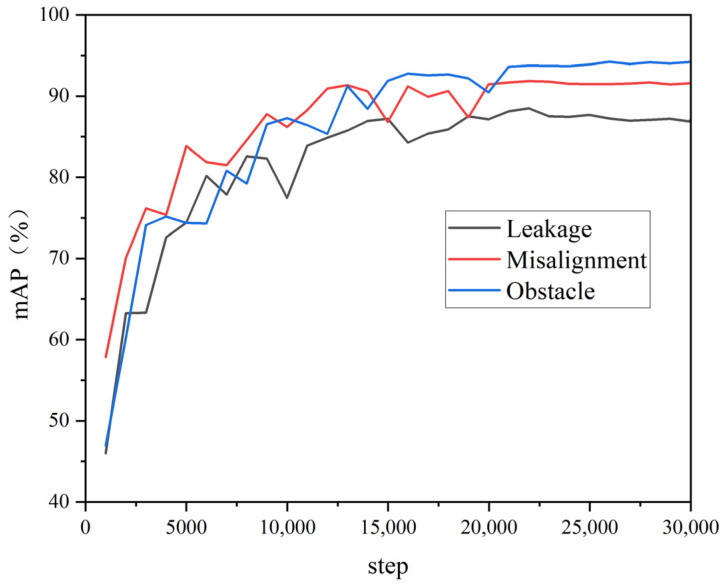
Impact of iteration count on the model.

After every 1000 iterations, mAP value testing was performed on the validation set to prevent overfitting, and AP50 was used as the evaluation metric for the COCO2017 dataset. AP50 refers to the average precision at an IoU threshold of 50%. The IoU is a metric used to measure the overlap between the predicted bounding boxes and ground-truth bounding boxes. Typically, when the IoU is greater than the threshold, the predicted bounding box is considered a correct prediction. The three curves in Figure 7 show the validation results for misalignments, obstructions, and cracks in the validation set. Table 3 shows the validation results after 5000, 10,000, 15,000, 20,000, 22,000, and 30,000 iterations. From Figure 7, it can be seen that when the number of iterations reached 22,000, the curves were stable. The mAP value of the trained model on the validation set was 0.914. After comprehensively considering the total loss during model training and the mAP value of the validation set, we chose the trained model with 22,000 iterations for subsequent research. 

#### 3.3.2. Comparison of Five Cases

To verify the impact of data augmentation, five experiments were conducted using four data augmentation methods (BigGAN, StyleGAN, StyleGAN2, and StyleGAN3). The validation set was used to validate the effectiveness of the trained models and the mAP values of each combination were tested. The experimental results are listed in Table 4. From Table 4, it can be observed that the data augmentation method using StyleGAN3 produced the best experimental results. This is because StyleGAN3 solves the problem of image coordinates and feature entanglement, achieving true image translation, rotation, and other invariances, thereby greatly improving the image synthesis quality. Therefore, StyleGAN3 generates more effective data, making it easier to extract the features of damaged areas during the data augmentation stage. Based on the segmentation effect of the trained model, we chose StyleGAN3 for the data augmentation.

### 3.4. Experimental Results

**Figure 8:**
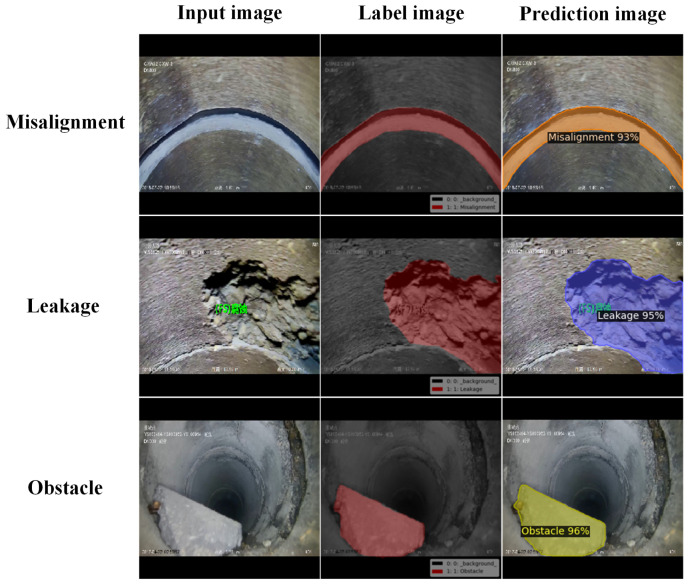
Example of model segmentation results under normal conditions.

**Figure 9:**
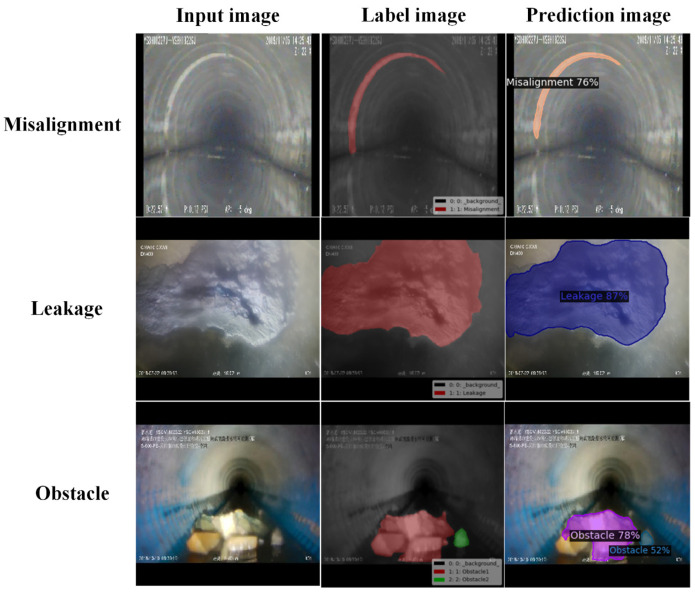
Example of model segmentation results under motion blur.

During image acquisition, motion blur may occur owing to the movement of the pipe robot or the fluid flow inside the pipe. Therefore, to verify the robustness and generalization ability of the trained model, experiments were conducted on normal and motion-blurred defect images. The test images in this section were not used for model training; that is, they were not included in the training set. In each case, three types of defects were tested: misalignment, obstruction, and crack. A total of 81, 57, and 75 images under normal conditions and 47, 32, and 17 images under motion-blurred conditions were used to test these three types of defects. The segmentation results for the two conditions are presented in Table 5. Examples of the test results are shown in Figure 8 and Figure 9, respectively. The figures show the input, label, and prediction results from left to right. The label images were manually annotated using the open-source LabelMe software, and the predicted images were the defect regions predicted by the Pipe-Sparse-Net model. From Table 5 and Figure 8 and Figure 9, it can be seen that under normal lighting conditions, the segmentation model was highly effective, with an mAP value of 0.916, and it also achieved good segmentation results under motion-blurred conditions, with an mAP value of 0.909.

Under motion-blurred conditions, the defect regions predicted by the Pipe-Sparse-Net model were less effective for cracks compared to other types of defects because the texture features of cracks are more complex and relatively difficult to extract under motion blur. However, the mAP value for defect segmentation using this model reached 0.882. Therefore, our trained Pipe-Sparse-Net model produced good segmentation results under the above test conditions, and almost all the defect regions could be segmented effectively. This demonstrates that the model is robust to noise and has strong generalization capability and stability. In future research, small segmentation errors could be reduced by improving the data collection devices and increasing the amount of data for specific defect types.

### 3.5. Comparative Experiment

**Figure 10:**
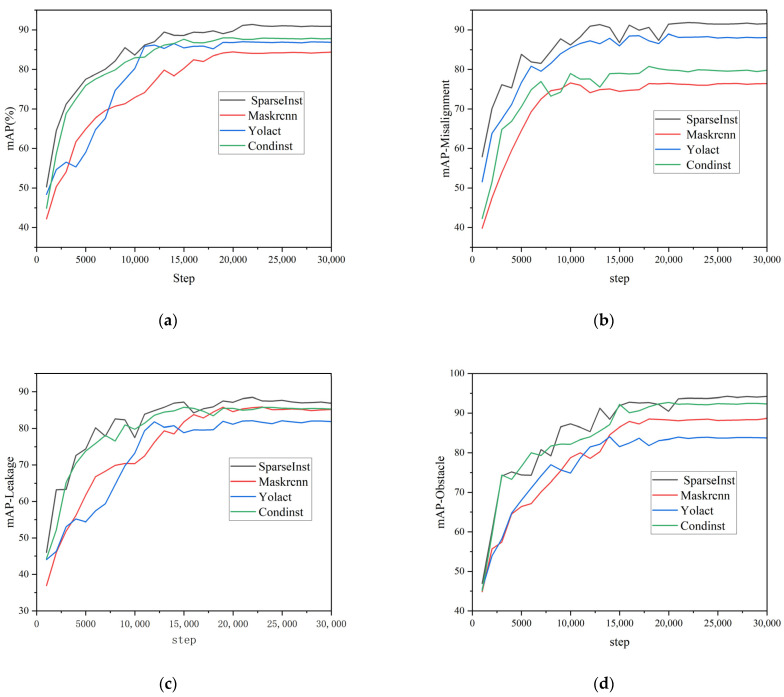
Comparison of training effects of four models: (**a**) all types of defects, (**b**) misalignment, (**c**) leakage, (**d**) obstacle.

**Figure 11:**
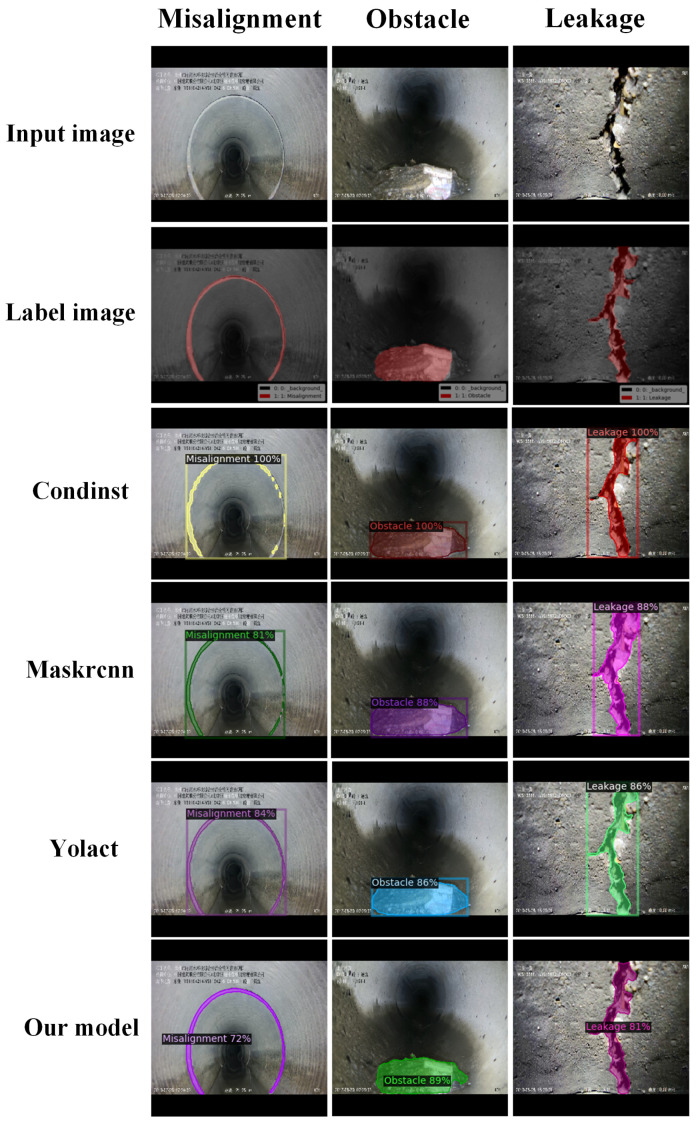
Examples of segmentation results from four models.

To compare the segmentation performance of Yolact, CondInst, Mask RCNN, and the proposed Pipe-Sparse-Net model on the same COCO2017 dataset, we conducted experiments under identical experimental conditions. The deep learning training platform used was equipped with a 12 GB GeForce RTX 3080 Ti GPU and 64 GB RAM I9 9900k. For three types of defects (misalignments, cracks, and obstructions), the mAP values of the four models were compared for three defect types: misalignments, cracks, and obstructions. From Table 6 and Figure 10a, it can be observed that the Pipe-Sparse-Net model accelerated by TensorRT achieved an mAP value of 0.914 for pipe defect segmentation. The mAP values for Yolact, Condinst, and Mask R-CNNs were 0.870, 0.880, and 0.844, respectively. From Figure 10b–d and Table 6, it can be seen that for the segmentation of misalignments, cracks, and obstructions, the segmentation accuracy of the proposed Pipe-Sparse-Net model is higher than those of the Yolact, Condinst, and Mask R-CNN models. The inference speed of the Pipe-Sparse-Net model accelerated by TensorRT increased by 44.64%. To further compare the segmentation effects of the four trained models, experiments were conducted on a test set that was not used for training or validation. Examples of the test results are shown in Figure 11. In the first column, the segmentation edges using Yolact, Condinst, and Mask R-CNNs were uneven. In the third column, the segmentation areas of Yolact, Condinst, and Mask R-CNN are significantly larger than the defect areas. Based on these results, the proposed Pipe-Sparse-Net model can detect defects with smooth and correct boundaries in pipe defect segmentation and has a better segmentation performance than traditional models.

## 4. Conclusions and Future Work

In previous research, deep learning-based pipeline defect detection algorithms have achieved high accuracy but have neglected the fact that robot motion or fluid flow in pipes can cause image blurring. To improve image quality and detection performance, this study proposes Pipe-Sparse-Net, an underground drainage pipeline defect segmentation system combined with StyleGAN3 for segmenting complex forms of defects in underground drainage pipelines. First, to address the problem of insufficient pipeline defect data, a clear image generation network called Pipe-Gan-Net, based on StyleGAN3, was established to increase the number of new defect images. Second, to improve pipeline defect segmentation accuracy and speed, a pipeline segmentation model called Pipe-Sparse-Net, based on SparseInst, was proposed to accurately predict drainage pipeline defect regions. To further improve the detection speed, an acceleration module called TensorRT was applied to the segmentation model, and extensive experiments demonstrated that the proposed method has good robustness, generalization capability, and stability for pipe defect segmentation under complex conditions. It also has a better segmentation performance than traditional models such as Yolact, Condinst, and Mask R-CNN. The proposed Pipe-Sparse-Net accelerated by TensorRT improved the inference speed by 44.64% compared with the unmodified network. This method provides a new approach for intelligent detection of underground pipeline defects.

The experimental results showed that after augmentation with StyleGAN3, the segmentation accuracy increased by 5.7%, demonstrating the efficacy of data augmentation in improving pipeline defect segmentation performance. Moreover, the proposed pipeline segmentation model, Pipe-Sparse-Net, achieves good segmentation performance on both motion-blurred and normal pipeline defect images, with accuracies of 91.6% and 90.9%, respectively. With the TensorRT module acceleration, the detection speed is increased by 44.64% to 56.7 fps, effectively solving the problem of achieving accurate and efficient pixel-level segmentation of pipeline defects. A comparative analysis with existing research results shows that the Pipe-Sparse-Net model accelerated by Tensor-RT achieves higher speed and accuracy than the Yolact, Condinst, and Mask R-CNN, with an increase in accuracy of at least 4% and a 45% improvement in segmentation speed. It has a clear advantage in the segmentation of pipeline defects and provides a new and effective method for the segmentation of underground drainage pipelines. However, it may be possible to further improve segmentation accuracy by enhancing boundary segmentation and introducing other types of sewer pipeline defects such as corrosion, foreign object intrusion, and illegal branch connections. In future, a database containing various pipeline defects should be established for automated detection. In addition, this study did not quantify defects, such as statistics on actual defect area sizes and numbers. We plan to improve this research in the future and propose a tracking network to count the number of defects in a pipeline segment. Our research goal in this field was to establish a complete pipeline defect detection and evaluation system.

## Figures and Tables

**Table 1:** Detailed distribution of different types of defects.

Category	Total	Training Set	Validation Set	Test Set
Misalignment	675	415	131	129
Obstacle	515	316	110	89
Leakage	395	247	57	91
Total	1585	978	298	309

**Table 2:** Hyperparameter settings.

Case	Learn Rate	Momentum	Weight Decay	mAP
1	1 × 10^−3^	0.9	5 × 10^−4^	0.906
2	5 × 10^−4^	0.8	1 × 10^−4^	0.907
3	5 × 10^−3^	0.85	5 × 10^−4^	0.905
4	1 × 10^−3^	0.8	1 × 10^−4^	0.911
5	1 × 10^−3^	0.9	1 × 10^−4^	0.901
6	5 × 10^−4^	0.9	5 × 10^−4^	0.914
7	5 × 10^−3^	0.9	5 × 10^−4^	0.910
8	5 × 10^−3^	0.8	1 × 10^−4^	0.904
9	5 × 10^−4^	0.85	5 × 10^−4^	0.896
10	1 × 10^−3^	0.85	1 × 10^−4^	0.909

**Table 3:** Validation results for different iteration counts.

Iterations	mAP			
	All	Misalignment	Obstacle	Leakage
5000	0.776	0.838	0.744	0.744
10,000	0.837	0.862	0.873	0.774
15,000	0.886	0.868	0.919	0.886
20,000	0.897	0.871	0.905	0.871
22,000	0.914	0.918	0.938	0.885
25,000	0.910	0.915	0.939	0.877
30,000	0.910	0.915	0.941	0.872

**Table 4:** Validation results of five cases (1. StyleGAN3 2. no augmentation 3. BigGAN 4. StyleGAN 5. StyleGAN2).

Case	mAP			
	All	Misalignment	Obstacle	Leakage
1	0.914	0.918	0.938	0.885
2	0.857	0.861	0.873	0.844
3	0.893	0.896	0.910	0.852
4	0.890	0.892	0.904	0.841
5	0.903	0.904	0.917	0.869

**Table 5:** Segmentation performance under different conditions.

Type	Normal Situation		Motion Blur	
	Number of Category	mAP	Number of Category	mAP
All	213	0.916	96	0.909
Misalignment	81	0.921	47	0.915
Obstacle	57	0.944	32	0.931
Leakage	75	0.888	17	0.882

**Table 6:** Comparison of segmentation performance among different models.

	With/Without Tensor RT	mAP	Speed
Our model	Yes	0.914	56.7 fps
Our model	No	0.909	39.2 fps
Yolact	No	0.870	35.9 fps
Condinst	No	0.880	24.7 fps
Mask R-CNN	No	0.844	15.4 fps

## Data Availability

Data are contained within the article.
